# Oscillating Evolution of a Mammalian Locus with Overlapping Reading Frames: An XLαs/ALEX Relay

**DOI:** 10.1371/journal.pgen.0010018

**Published:** 2005-08-12

**Authors:** Anton Nekrutenko, Samir Wadhawan, Paula Goetting-Minesky, Kateryna D Makova

**Affiliations:** 1 Department of Biochemistry and Molecular Biology, Pennsylvania State University, University Park, Pennsylvania, United States of America; 2 Center for Comparative Genomics and Bioinformatics, Pennsylvania State University, University Park, Pennsylvania, United States of America; 3 The Huck Institutes for Life Sciences, Pennsylvania State University, University Park, Pennsylvania, United States of America; 4 Department of Biology, Pennsylvania State University, University Park, Pennsylvania, United States of America; National Institute of Genetics, Japan

## Abstract

XLαs and ALEX are structurally unrelated mammalian proteins translated from alternative overlapping reading frames of a single transcript. Not only are they encoded by the same locus, but a specific XLαs/ALEX interaction is essential for G-protein signaling in neuroendocrine cells. A disruption of this interaction leads to abnormal human phenotypes, including mental retardation and growth deficiency. The region of overlap between the two reading frames evolves at a remarkable speed: the divergence between human and mouse ALEX polypeptides makes them virtually unalignable. To trace the evolution of this puzzling locus, we sequenced it in apes, Old World monkeys, and a New World monkey. We show that the overlap between the two reading frames and the physical interaction between the two proteins force the locus to evolve in an unprecedented way. Namely, to maintain two overlapping protein-coding regions the locus is forced to have high GC content, which significantly elevates its intrinsic evolutionary rate. However, the two encoded proteins cannot afford to change too quickly relative to each other as this may impair their interaction and lead to severe physiological consequences. As a result XLαs and ALEX evolve in an oscillating fashion constantly balancing the rates of amino acid replacements. This is the first example of a rapidly evolving locus encoding interacting proteins via overlapping reading frames, with a possible link to the origin of species-specific neurological differences.

## Synopsis

One of the possible ways to achieve tight co-expression of two proteins is to encode them within a single mRNA. The *GNAS1* gene in mammals does just that: it encodes two interacting signaling polypeptides within a single transcript using nested reading frames shifted one nucleotide relative to each other. The exceptionally high GC content of the region where the two reading frames overlap diminishes the probability of encountering stop codons but makes the locus highly mutable. To preserve their ability to interact functionally with each other despite the high mutation rate, the two polypeptides appear to evolve in an oscillating fashion, trying to maintain approximately equal rates of amino acid substitutions. This unexpected observation provides new insights into the evolution of mostly overlooked overlapping coding regions in eukaryotic genomes.

## Introduction

The *GNAS1* locus encodes the stimulatory G-protein subunit α, a key element of the classical signal transduction pathway linking receptor–ligand interactions with the activation of adenylyl cyclase and a variety of cellular responses [1–3]. The gene is subject to complex imprinting, producing a spectrum of maternally, paternally, and biallelically derived transcripts [4]. The major paternally imprinted transcript of the gene is expressed primarily in neuroendocrine tissues and includes an unusually large upstream exon (the XL-exon) comprising over 50% of the protein-coding region. The XL-exon contains two completely overlapping reading frames in the same orientation but shifted one nucleotide relative to each other so that codon positions 1, 2, and 3 of the first frame overlap with positions 3, 1, and 2 of the second frame. In humans the first frame of the exon encodes 388 N-terminal amino acids of a 736-residue extra large form of Gα (XLαs) [5–10]. The second frame encodes all 322 amino acids of alternative gene product encoded by the XL-exon (ALEX) and terminates exactly at the end of the exon. The internal section of the XL exon contains imperfect repeated units of variable length translated into amino acid repeats averaging 13 residues in both XLαs and ALEX [4]. The repeat number varies in a studied human population (*n* = 276), with the majority carrying a 13-unit allele, while an insertion of an additional unit (the 14-unit allele) is found in 2.2% of surveyed individuals [11]. Heterozygous individuals with a maternally inherited 14-unit allele and 13-unit homozygotes are normal. Conversely, carriers of a paternally inherited 14-unit allele exhibit hyperactivity of the G-protein pathway and suffer from a variety of pathological conditions such as mental retardation, brachymetacarpia, hypertrichosis, hypotonia, growth deficiency, or prolonged trauma-induced bleeding [12]. Binding assays showed a decreased affinity between XLαs and ALEX in individuals carrying the 14-unit allele that leads to an elevated concentration of free XLαs (unbound to ALEX) capable of activating adenylyl cyclase [12]. As a result, the intracellular cAMP concentration rises to over 600% of the normal level. Thus, ALEX regulates the intracellular cAMP level by specifically binding XLαs and preventing it from interacting with the receptors and adenylyl cyclase [12,13]. Loss-of-function mutations involving XLαs also lead to severe adverse effects. Mice lacking XLαs expression show poor postnatal development with the majority dying within 48 h of birth [5].

The functional importance of XLαs and ALEX suggested by these examples implies that this locus should be under considerable selective constraint. Yet the XL-exon evolves at a remarkable pace: the nucleotide identity between human and mouse XL-exon is only 71%, and the amino acid identity between human and mouse ALEX is 53% [13]. For comparison, the average nucleotide and amino acid identities between human and mouse protein-coding genes and their protein products are 86% and 89%, respectively [14]. Why would a locus encoding two essential signaling proteins evolve so rapidly?

## Results/Discussion

To take a closer look at the evolutionary dynamics of XLαs and ALEX, we sequenced the XL-exon from eight primates and immediately found striking differences within the repeat-containing region (we used XL-exon boundaries as described in Hayward et al. [4]; also see Methods). All studied species that included human, apes (chimpanzee, gorilla, orangutan, and gibbon), Old World monkeys (colobus and macaque), and a New World monkey (squirrel monkey) varied in the number and/or sequence of repeated units (Figure 1). Human had the smallest number of repeats, while the remaining taxa contained at least one additional repeat unit between positions I and N (Figure 1), a region where an insertion in humans is linked to disease. Taxa closest to human (chimpanzee, gorilla, and orangutan) carried the largest number of repeat units and an additional insertion at position B. Gibbon, colobus, macaque, and squirrel monkey contained an additional insertion at position H. Assuming that the sequenced alleles are fixed in the respective primate populations, XL-exon experienced an episode of repeat expansion in the greater ape lineage followed by a dramatic repeat loss on the branch leading from the human/chimpanzee ancestor to modern humans (Figure 2). Note that in all sampled species both reading frames remain intact regardless of the insertion/deletion events. The observed pattern may have implications for the evolution of species-specific neurological and metabolic differences (discussed below) since the variation in the number of repeats has profound developmental and physiological effects [5,11,12].

**Figure 1:**
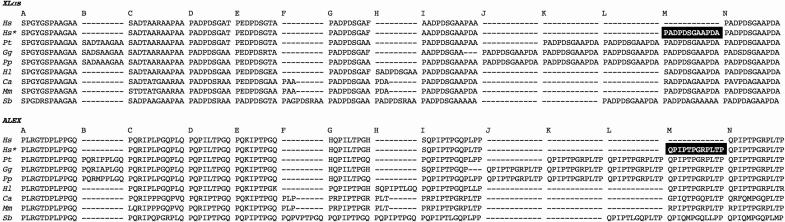
Alignment of Internal Repeat Region in XLαs and ALEX Polypeptides Black boxes highlight the position of the disease-linked repeat in the 14-unit human allele (Hs*). Sequences upstream and downstream of the shown region can be aligned unambiguously. Species abbreviations as follows: Hs, *Homo sapiens* (human); Pt, *Pan troglodytes* (chimpanzee); Gg, *Gorilla gorilla* (gorilla); Pp, *Pongo pygmaeus* (orangutan); Hl, *Hylobates lar* (gibbon); Ca, *Colobus angolensis* (colobus monkey); Mm, *Macaca mulatta* (macaque); Sb, *Saimiri boliviensis* (squirrel monkey).

**Figure 2:**
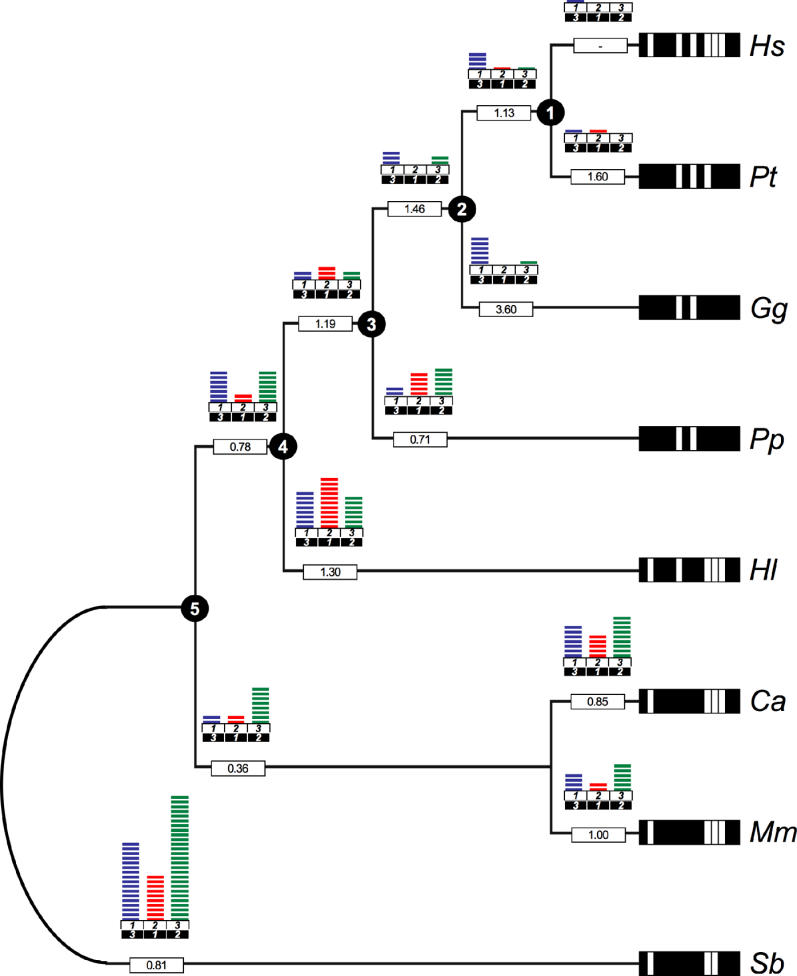
Evolutionary Oscillation between XLαs and ALEX Frames Revealed from Nucleotide Substitution Analysis The ratio of maximum likelihood estimates of nonsynonymous rates between the two frames (^XL^ *K*_A_/^ALEX^ *K*_A_) is shown on each branch. A series of colored bars above each branch shows the number of nucleotide substitutions at each codon position reconstructed using parsimony. Each bar represents a single substitution. The codon positions are numbered as follows: black letters on white background (XLαs frame); white letters on black background (ALEX frame). Boxes at the ends of external branches show repeat structure of the XL-exon in each species (white = deletion). Species abbreviations are as in Figure 1.

Next, we analyzed the pattern of nucleotide substitutions within the XL-exon (excluding the repeat-containing region) and observed a striking oscillation of amino acid replacement rates between the XLαs and ALEX. The interaction between the two proteins imposes a unique constraint: if one protein changes the other needs to rapidly “evolve” a compensatory substitution to preserve the mutual affinity. Although this cannot be observed directly in our data because such changes are likely to occur within each lineage in rapid succession, the overall effect of this process should result in similar rates of amino acid replacements in the two proteins. To test this hypothesis we compared nucleotide substitutions between XLαs and ALEX frames in sequenced species. Classical measures of nucleotide substitution rates such as *K*_S_ and *K*_A_ [15] are not directly applicable here because of the interdependence of the two overlapping frames [16–18]. However, these measures can be used in a relative context. Specifically, the ratio of nonsynonymous rates between the two frames (^XL^ *K*_A_/^ALEX^ *K*_A_) can be used to test the equality of amino acid replacement rates between the two proteins. To carry out this analysis we reconstructed a phylogenetic tree using unambiguously aligning portions of the XL-exon. For every branch of the tree we computed the ^XL^ *K*_A_/^ALEX^ *K*_A_ ratio using maximum likelihood estimates of nonsynonymous rates for each frame (Figure 2). Ratios vary considerably among branches. For example, branches originating from node 3 (3→Pp and 3→2) show opposing ^XL^ *K*_A_/^ALEX^ *K*_A_ ratios. However, none of the ratios is significantly different from 1 (*p-*values from Fisher's exact test are between 0.14 and 0.77), supporting our hypothesis that the two proteins constantly co-evolve and maintain ^XL^ *K*_A_/^ALEX^ *K*_A_ of approximately 1.

A possible caveat of this analysis is the use of internal nodes because the likelihood method we employed to estimate branch-specific rates was not intended to handle coding sequences with multiple reading frames. To address this, we estimated pairwise *K*_A_ between XLαs and ALEX reading frames. For this purpose we developed a neighbor-dependent modification of the Nei–Gojobori (NG) method [19]. Unlike the classical NG, our method estimates the number of synonymous and nonsynonymous changes in a given frame (i.e., XLαs) without considering any pathways that would create nonsense codons in the other frame (i.e., ALEX). The resulting estimates were only slightly different from the NG, Yang-Nielsen [20], and likelihood [21] methods, as the high GC content of XL-exon (68% in human) decreases the chance of encountering pathways that contain nonsense codons (Table 1). We used the new *K*_A_ estimates to calculate the ^XL^ *K*_A_/^ALEX^ *K*_A_ ratio for each pair of species. Again, although the ratios varied substantially, none was significantly different from 1 (at 1% level; Table 1). The observed oscillation of the ^XL^ *K*_A_/^ALEX^ *K*_A_ ratio around 1 likely implies constant adjustment between the two proteins aimed at maintaining mutual affinity.

**Table 1:**
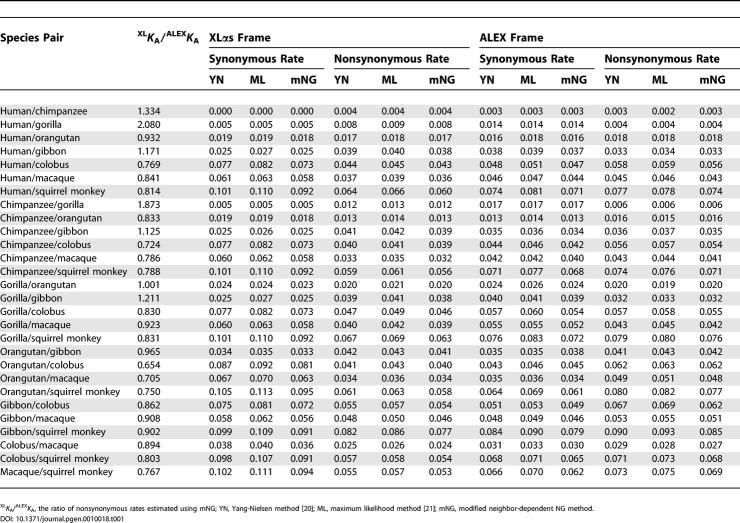
Pairwise Synonymous and Nonsynonymous Rates in XLαs and ALEX Protein-Coding Regions

^XL^ *K*_A_/^ALEX^ *K*_A_, the ratio of nonsynonymous rates estimated using mNG; YN, Yang-Nielsen method [20]; ML, maximum likelihood method [21]; mNG, modified neighbor-dependent NG method.

The phenomenon of oscillation is also confirmed by the pattern of nucleotide substitutions at different codon positions. Third codon positions of the XLαs frame, where most changes are synonymous, correspond to second codon positions of the ALEX frame where all substitutions lead to amino acid replacements. Similarly, third codon positions of the ALEX frame overlap with first codon positions of the XLαs where most substitutions are nonsynonymous. To visualize the substitution process at the level of codon positions, we used maximum parsimony to reconstruct ancestral sequences at the internal nodes of the tree in Figure 2. We modified the original parsimony algorithm by omitting ancestral states that may create stop codons in either of the two frames. Although ancestral sequences reconstructed using parsimony cannot be used as observed data [22], this analysis once again shows evolutionary fluctuation between the two frames (Figure 2). For example, the majority of substitutions on branches leading to Ca, Mm, and Sb are in the third codon position of the XLαs frame (corresponding to the 0-fold degenerate second codon position of the ALEX frame). This is also the case for the branch leading to Pp, while other branches within the human/ape clade show the opposite pattern—most substitutions are now in mostly 0-fold degenerate first and second codon positions of the XLαs frame. In addition, there are examples of recurrent substitutions leading to the same amino acids in different lineages (Table 2), thus, suggesting that multiple optimal variants of the two proteins are allowed.

**Table 2:**
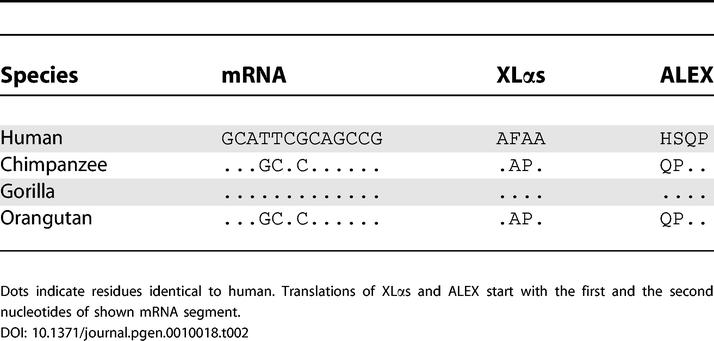
An Example of Recurrent Substitutions in Human and Apes

Dots indicate residues identical to human. Translations of XLαs and ALEX start with the first and the second nucleotides of shown mRNA segment.

The high GC content of the XL-exon (ranging from 68% in human to 71% in squirrel monkey) is “the blessing and the curse” of the locus: it appears to be required for the maintenance of the two reading frames, but inevitably leads to a high substitution rate. A consequence of the high GC content is the abundance of GC-rich codons in the XLαs and ALEX frames. For instance, the most abundant codons in XLαs and ALEX frames are GCC (10.6%) and CCG (8.9%), respectively (Figure 3). For comparison, average frequencies of these codons in humans (estimated from RefSeq genes) are 2.8% and 0.7%, respectively. The GC content may be driven up by a selection acting against mutations to A and T, as these can lead to the formation of stop codons (TAA, TAG, TGA) in either of the two frames. To test this hypothesis, we simulated the eight sequences in our dataset using three different codon frequency tables compiled from (1) all human RefSeq genes, (2) XLαs reading frame, and (3) ALEX reading frame. All other parameters (phylogenetic tree, branch lengths, transition/transversion ratio, codon number, and the *K*_A_/*K*_S_ ratio as estimated from the original dataset) were fixed, and each simulation was performed 1,000,000 times. Each set of simulated sequences was examined for the presence of alternative reading frames. For example, for every set of sequences simulated using XLαs codon frequencies, we looked for the presence of an alternative reading frame in +1 phase. None of the sets from the first simulation (RefSeq codon frequencies) contained such frames, whereas approximately 1% of sets in each of the second (XLαs codon frequencies) and the third (ALEX codon frequencies) simulations contained alternative frames in +1 and −1 positions, respectively. Thus the high GC content allows for overlapping reading frames.

**Figure 3:**
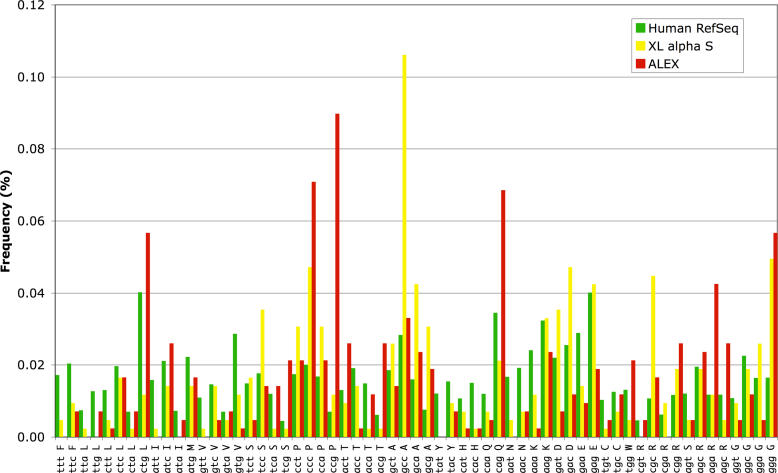
Codon Usage Estimated from Human RefSeq Genes, XLαs Coding Region, and ALEX Coding Region Green indicates human RefSeq genes, yellow indicates XLαs coding region, and red indicates ALEX coding region.

The high GC content also leads to an excess of CpG dinucleotides, which occupy approximately 20% of XL-exon (108–119 CpG sites or 18%–21% of the sequence length, depending on the species). This is significantly higher than in the majority of primate sequences (empirical *p* = 0.0013): the proportion of CpG sites in human protein-coding regions from the RefSeq database have narrow distribution with a mean of 7% (99% confidence interval: [7.17%; 7.43%]). In mammals, mutation rate at CpG dinucleotides is 10–20 times higher than at other sites [23–25]. As a result, although CpG sites occupy only approximately 20% of the sequence in our dataset, approximately 50% of the observed nucleotide substitutions (responsible for approximately 30% of amino acid replacements) occur at these sites (Table 3). In this analysis we do not correct for multiple substitutions because existing models cannot be used in the context of XLαs/ALEX locus. Thus, the actual rate of evolution of XL-exon is even higher than observed. Remarkably, the majority of potential deamination events at CpG sites (CpG → CpA and CpG → TpG transitions) do not create stop codons in either of the two reading frames. Indeed, the in silico deamination of all CpG sites (109–118 replacements, depending on the species) to either TpG or CpA created only four stop codons in the XLαs and none in the ALEX frame in each species. In contrast, the simulated deamination caused on average 140 and 129 amino acid changes in XLαs and in ALEX, respectively. Therefore, high GC content leads to the high intrinsic mutability of the XL-exon but allows avoidance of stop codons.

**Table 3:**
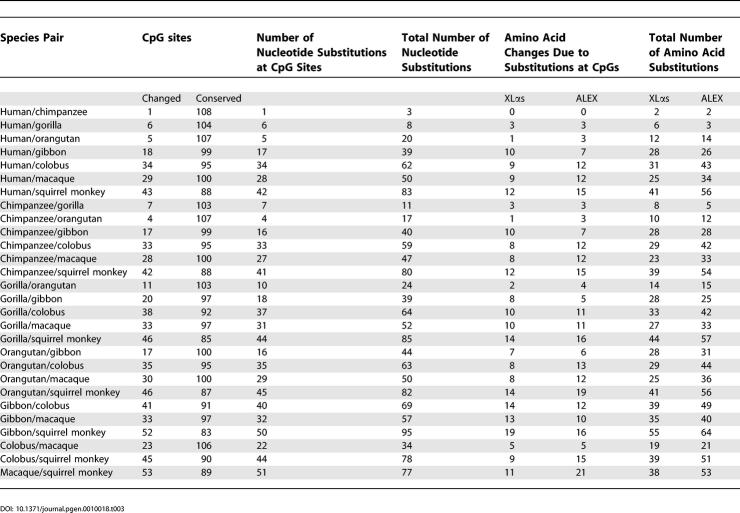
Nucleotide and Amino Acid Replacements at CpG Sites

These results suggest the following model of XLαs/ALEX evolution that favors purifying selection acting on the two proteins. The benefit in encoding the two signal transduction proteins within the same mRNA molecule might be the tight expression coupling: it guarantees that the two proteins are made at the same place and at the same time. To maintain two long, overlapping reading frames the XL-exon must contain an excess of GC-rich codons, but this also leads to the elevated frequency of mutation-prone CpG dinucleotides. Because the two proteins physically interact, they must accumulate amino acid substitutions in concert: neither can change too much relative to the other as their mutual affinity may become adversely affected. Therefore, a nonsynonymous mutation causing a deleterious change in affinity must be quickly corrected by either reversal or compensatory change [26]. The high mutation rate of the XL-exon, which is due to the high frequency of CpG sites, may allow such “corrective” changes to occur quickly. The reversals and/or compensatory changes likely occur in rapid succession, keeping the overall ratio of nonsynonymous changes (^XL^ *K*_A_/^ALEX^ *K*_A_) close to 1 for a given lineage, a phenomenon observed in our data (see Figure 2). The shortcoming of this stochastic process is that by constantly adjusting to each other, XLαs and ALEX may drift beyond the acceptable level of mutual affinity. One way to overcome this situation might be by changing the number of internal repeat units that may serve as sandbags on an air balloon—allowing rapid changes in affinity in a single step (e.g., an addition/deletion of a single repeat unit in humans causes a significant change in affinity [12]). This may explain remarkable variation in the number of internal repeat units in human and apes. This simple model implies that the two proteins evolve under a purifying selection scenario and that the observed high substitution rate is a consequence of the high GC content imposed by the need to maintain two reading frames.

We cannot rule out an alternative adaptive evolution explanation of the variation in the number of repeats and the pattern of amino acid changes in XLαs and ALEX. XLαs and ALEX are predominantly expressed in neuroendocrine tissues where they likely play a role in the development and maintenance of neurological functions [5,12,27]. In particular, XLαs expression is evident in distinct regions of the brain controlling processing of sensory information (locus coeruleus) and innervation of orofacial muscles (i.e., facial nucleus) [5]. Individuals with disrupted XLαs/ALEX interactions have multiple neurological complications, including feeding motility problems, psychomotor retardation, and disturbed behavior [12]. It is therefore plausible that amino acid replacements and the variation in the internal repeat number may have been associated with the adaptation of G-protein signaling to specific neurological functions, perhaps specific to humans. However, to reliably distinguish between the possibilities of purifying and positive selection, it is necessary to experimentally measure XLαs/ALEX affinities in primates—a direction currently pursued by our laboratories.

Is the XLαs/ALEX locus the only example of extensively overlapping reading frames in mammals? Only three additional cases are known where protein products of both reading frames were biochemically characterized. These include genes for the cyclin D-dependent kinase inhibitor INK4a [28], X-box protein 1 [29], and a region of overlap between 4E-BP3 and MASK [30]. Discovery of genes with alternative reading frames is hampered by our disbelief in their existence. For example, *ALEX* was discovered long after the *XLαs* gene had been identified [9,13]. Early results from our laboratories indicate that there are many more genes (possibly hundreds) potentially encoding multiple proteins via alternative reading frames. In each case the alternative reading frame is conserved in all known mammalian orthologs of a gene. Similarly to *XLαs,* most of these genes have been known for some time but the presence of the alternative reading frame has never been discovered. Biochemical characterization of these alternative products is underway and may assist us in discerning yet another facet of mammalian gene organization and evolution.

## Materials and Methods

### Amplification and sequencing of XL-exon.

The entire XL-exon was amplified from genomic DNA in all eight species, using primers 990F and 2954R or 2428R (Table 4). These primers were designed using published human sequence [4]. Specifically positions 318 and 511 within XL-exon were considered to be starts of XLαs and ALEX coding regions, respectively (as defined in [31] and [13]). PCR conditions were as follows: 1.75 U Taq (Expand High Fidelity PCR System; Roche Diagnostics, Mannheim, Germany), 0.2 mM dNTPs, 300 nM of each primer, 1 ng/μl template DNA, PCR buffer with MgCl_2_ (Expand High Fidelity PCR System), and 7% DMSO. Hot start reaction was carried out using an ABI Thermocycler 9700 (Applied Biosciences, Foster City, California, United States) under the following conditions: 94 °C for 5 min (initial denaturation), followed by 30 cycles of denaturation at 94 °C for 30 s, annealing at 61 °C for 30 s, elongation at 72 °C for 2 min, and final extension at 72 °C for 5 min. The amplified products were purified using the QIAquick PCR purification kit (Qiagen, Valencia, California, United States). In each taxon amplification products were sequenced in both directions using species-specific primers (Table 4). Sequencing reactions were carried out using 1 μM of primers, 7% DMSO, 35–50 fmol of template DNA, and CEQ DTCS Quick Start Kit (Beckman Coulter, Allendale, New Jersey, United States) in an ABI Thermocycler 9700 under the following conditions: 40 cycles of 96 °C for 20 s, 50 °C for 20 s, and 60 °C for 4 min. Traces were obtained using Beckman Coulter CEQ 8000 sequencer. Sequence traces were manually analyzed using the DNAStar software package (http://www.dnastar.com/web/index.php).

**Table 4:**
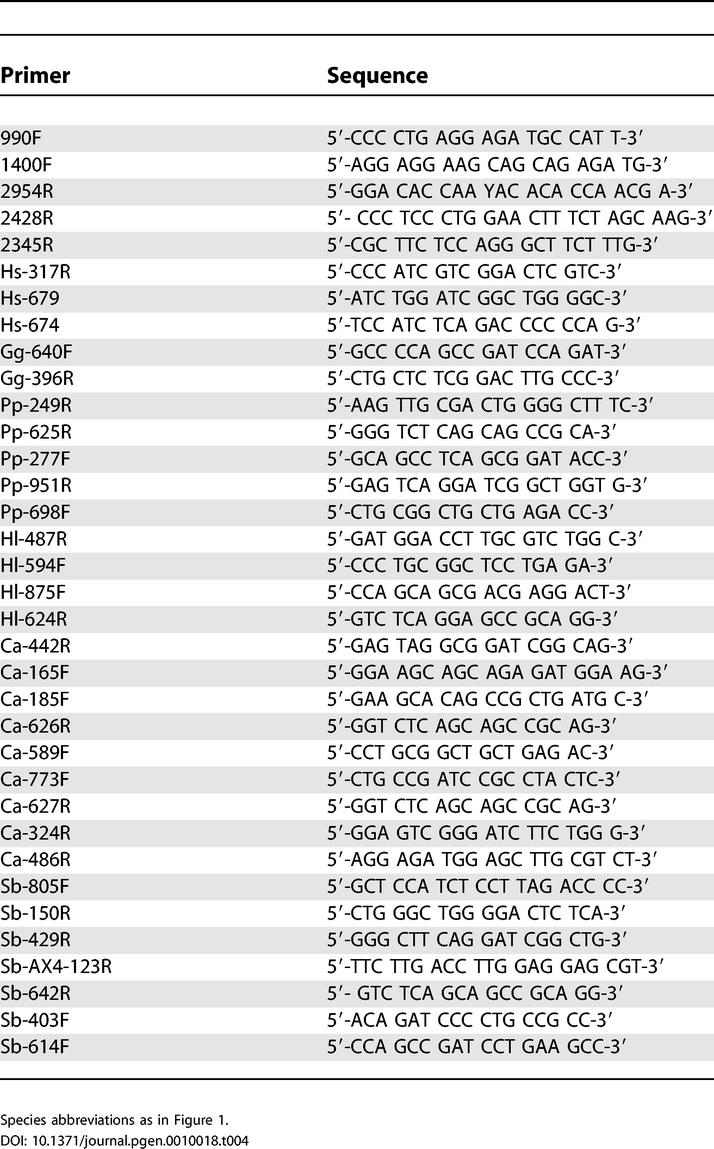
Amplification and Sequencing Primers

Species abbreviations as in Figure 1.

### Data analysis.

Reliable alignment was generated by first translating nucleotide sequences from each taxa, aligning the translations using ClustalW [32], refining these alignments manually, and then reconstructing nucleotide alignments, using the protein alignment as a guide. Phylogenetic tree and most statistics were calculated using the PAML software package [33]. All analyses were performed on the region of overlap between the two reading frames, excluding the repetitive region. Synonymous and nonsynonymous rates were apportioned among the branches of the tree using the codeml program of the PAML package under the free ratio model [34].

The neighbor-dependent modification of the NG method was written in PERL programming language and is available from the authors upon request. The only difference from the classical NG algorithm [19] is that pathways creating stop codons in the alternative reading frame are ignored by our method. For example, let us consider the alignment in Table 5.

**Table 5:**
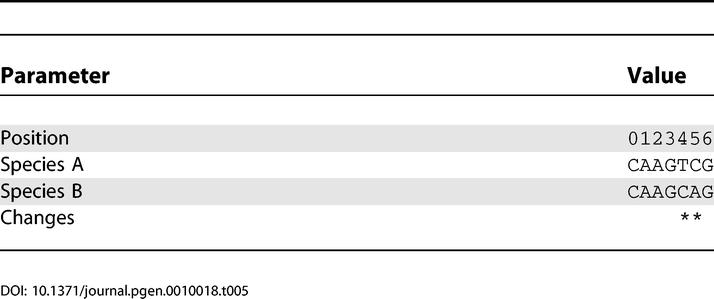
Sample Alignment Parameters for Neighbor-Dependent Modification of the NG Method

The alignment contains two reading frames: frame 0 starting at position 0 and frame 1 starting at position 1. The second codon of frame 0 contains two substitutions, and so there are two possible parsimonious pathways:


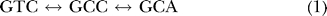



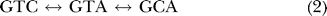


Pathway 2 would convert the second codon of frame 1 into a stop (TAG), and so it is not considered by our method.

To test whether the GC content of the XL-exon is required for the coexistence of the two reading frames, we first estimated codon frequencies in (1) human RefSeq genes, (2) XLαs reading frame, and (3) ALEX reading frame. This procedure was performed using a custom-designed PERL script. Coding regions of human RefSeq genes were downloaded from the National Center for Biotechnology Information ftp site (ftp://ftp.ncbi.nlm.nih.gov). We then used the evolver program of the PAML package to simulate 1,000,000 sequence sets, using the three codon frequency tables. Each set contained eight sequences corresponding to primate species used in this study. All other parameters accepted by evolver (phylogenetic tree, branch lengths, transition/transversion ratio, codon number, and the *K*_A_/*K*_S_ ratio) were taken from codeml output generated during nucleotide substitution analysis of our data and were fixed in all three simulations. Each set of simulated sequences was then inspected for the presence of +1 and −1 overlapping reading frames. A set of simulated sequences was considered to have an overlapping reading frames if such frame was greater than or equal to 1,000 bp and was conserved in all eight sequences within the set.

Analysis of substitutions at CpG sites was carried out using a collection of PERL script, which can be obtained upon request.

## Supporting Information

### Accession Numbers

Sequences reported in this paper have been deposited in GenBank (http://www.ncbi.nlm.nih.gov/Genbank) under the following accession numbers: *Homo sapiens* (AJ224868), *Colobus angolensis* (AY771990), *Gorilla gorilla,* *Macaca mulatta,* *Saimiri boliviensis,* *Homo sapiens,* and *Pan troglodytes* (AY898801–AY898805), *Hylobates lar* (AY4787144), and *Pongo pygmaeus* (AY787145).

## Abbreviations

ALEX: alternative gene product encoded by the XL-exon
NG: Nei–Gojobori
XLαs: extra large form of Gα
